# Analysis of Positive Results of ^18^F-FDG PET/CT Imaging after Hematopoietic Stem Cell Transplantation in Lymphoma

**DOI:** 10.3390/diagnostics13122027

**Published:** 2023-06-11

**Authors:** Na Dai, Rongcui Cai, Shengming Deng, Shibiao Sang

**Affiliations:** Department of Nuclear Medicine, The First Affiliated Hospital of Soochow University, Suzhou 215006, China; daina0531@suda.edu.cn (N.D.); cairongcuisdfyy@163.com (R.C.)

**Keywords:** lymphoma, SCT, post-transplantation ^18^F-FDG PET/CT, Deauville score, false-positive FDG uptake

## Abstract

Purpose: The purpose of this study was to differentiate between false-positive and true-positive positron emission tomography (PET) results after hematopoietic stem cell transplantation (SCT) for lymphoma involvement by analyzing several clinical variables and specific imaging features. Patients and Methods: Patients with lymphoma who received SCT and underwent post-transplantation ^18^F-FDG PET/CT scans between January 2013 and April 2021 at our institution were included. Associations between PET positivity and related clinical information were assessed using *t*-tests and χ^2^ tests. The significance of variables differentiating benign lesions from malignant FDG-avid lesions was evaluated by logistic regression analysis. Survival probabilities were derived from Kaplan-Meier curves and compared using the log-rank test. Results: A total of 185 patients (235 post-transplantation PET/CT scans) were enrolled in our present study. Compared with those with true-positive PET results, patients with false-positive PET results exhibited a better prognosis. For the autologous SCT group, false-positive cases were more commonly seen when FDG-avid foci appeared outside the sites of the original disease (*p* = 0.004), and the integrated CT imaging showed negative results (*p* = 0.000). In multivariate logistic regression analysis, integrated CT results were the only significant factor. For the allogeneic SCT group, false-positive cases were significantly more commonly seen when DS = 4 (*p* = 0.046), FDG-avid foci appeared outside the sites of the original disease (*p* = 0.022), and the integrated CT imaging showed negative results (*p* = 0.001). In a multivariate logistic regression analysis, whether FDG-avid foci were in the sites of the original disease and integrated CT results were both significant factors. Conclusion: False-positive FDG uptake in post-transplantation PET was not uncommon. Several variables could provide an important reference to differentiate false-positive from true-positive post-SCT PET results for lymphoma involvement. Trial registration number: ChiCTR2300067355.

## 1. Introduction

Worldwide, about 0.6 million new cases of lymphoma occurred in 2020, including 83,087 new cases of Hodgkin’s lymphoma (HL) and 544,352 new cases of non-Hodgkin’s lymphoma (NHL) [1]. The cure rate for lymphoma has improved in recent years with the application of hematopoietic stem cell transplantation (SCT) [2,3]. Approximately half of the patients can achieve long-time survival after SCT [4,5]. It is undoubtedly critical to identify the efficacy of transplantation at an early stage. Moreover, 2-deoxy-2-[^18^F]fluoro-D-glucose positron emission tomography/computed tomography (^18^F-FDG PET-CT) has been widely used for staging, restaging, aggressiveness evaluation, and response monitoring of lymphoma since PET-CT was incorporated into the guidelines for lymphoma in 2007 [6,7]. Our preliminary studies [8,9] have shown that post-transplantation PET is helpful for the prognostic assessment of lymphoma patients.

However, one disadvantage of ^18^F-FDG PET in diagnosing lymphoma is that it is sometimes difficult to distinguish whether the FDG-avid foci are lymphoma involvement or inflammatory changes [10,11,12] because the accumulation of ^18^F-FDG reflects glucose metabolism of both cancer cells and immunologically competent cells in the tumor [13]. Another previous report has shown that filgrastim administration may cause false-positive findings in the liver with ^18^F-FDG PET [14]. Considering individual immune function and tumor heterogeneity (including inter- and intra-patient tumor heterogeneity), patients differ in their immunological responses to the therapy. To minimize the frequency of these potentially confounding findings [15,16,17], it is suggested that PET scans should not be performed for at least 3 weeks, and preferably 6 to 8 weeks, after completion of therapy [7]. High-dose chemotherapy or radiotherapy before SCT leaves patients in a state of temporary immunodeficiency, making them more prone to inflammatory or infectious lesions. These reasons may cause uncertain interpretation of ^18^F-FDG PET/CT imaging results after transplantation. Previous studies have shown a high rate of false-positive PET after transplantation [18], while the role of post-SCT PET in the evaluation of efficacy or prognosis remains controversial [19,20]. Whether this high rate of false positives is related to the above controversy is still unknown.

In the present study, we aimed to differentiate between false-positive and true-positive post-SCT PET results for lymphoma involvement by analyzing several clinical variables and specific imaging features and optimizing the interpretation of post-SCT ^18^F-FDG PET/CT images.

## 2. Material and Methods

### 2.1. Study Design

The inclusion criteria were as follows: (1) pathologically confirmed lymphoma (including NHL and HL); (2) received SCT between January 2013 and April 2021 at the First Affiliated Hospital of Soochow University; (3) underwent post-transplantation ^18^F-FDG PET/CT scans within 6 months after auto-SCT or within 12 months after allo-SCT. The exclusion criteria were: (1) patients who received systemic therapy for lymphoma other than maintenance therapy within the time window between day 0 of transplantation and post-transplantation PET. (2) Patients who had an incomplete follow-up. Status before and after transplantation was determined according to the International Working Group criteria (IWGc) [6,7,21].

Indications for HSCT (allogeneic or autologous) vary by disease type and remission status according to disease-specific NCCN Guidelines [22,23,24]. In our institution, lymphoma patients who received transplantation generally meet the following conditions: autologous SCT can be safely used for patients ≤75 years old, generally in good condition and without obvious organ function damage or comorbidities. Myeloablative conditioning allogeneic SCT can be used for patients ≤55 years old. Patients between 65–70 years old with hematopoietic cell transplant-composite risk (HCT-CI) ≤4 could consider receiving allogeneic SCT with reduced-intensity/nonmyeloablative conditioning(RIC/NMA).

We calculated the diagnostic efficiency of ^18^F-FDG PET and integrated CT. ^18^F-FDG PET/CT results were compared with the findings of the pathological examination and follow-up practice (more than 6 months). Overall survival (OS) was defined as the interval from day 0 of SCT until the time of death from any cause or last follow-up.

Approval was obtained from the institutional review board of the First Affiliated Hospital of Soochow University. The trial registration number was ChiCTR2300067355. The requirement for written informed consent from patients was waived.

### 2.2. ^18^F-FDG-PET/CT Imaging

All patients underwent whole-body ^18^F-FDG PET/CT on a GE Discovery STE16 PET/CT (GE Medical systems, Milwaukee, WI, USA). All patients had fasted for at least 6 h, and their blood glucose levels were less than 11 mmol/L before injection. Whole-body PET images (from skull base to mid-thigh) were acquired approximately 60 min after injection of ^18^F-FDG (0.11–0.14 mCi/kg). Emission data were acquired for approximately 2 min in each bed position, with an average of 7–10 bed positions per scan. CT examinations were obtained with the following scan parameters: 3.5 mm/slice, 140 kV, 120 mA. PET data were reconstructed with CT-based attenuation correction by using an iterative algorithm. 

Patients were assigned into 2 groups based on the results of post-SCT PET using the Deauville 5-point scale [7,25]: (1) the negative PET group, DS < 4; (2) the positive PET group, DS 4 or 5, which could not be attributed to a physiologic or inflammatory cause.

According to the CT-Based Response on the Lugano Classification, patients were divided into two 2 groups: (1) the negative CT group, target nodes/nodal masses that regressed to ≤1.5 cm in the longest transverse diameter of a lesion (LDi); (2) the positive CT group, target nodes/nodal masses still >1.5 cm in LDi or/and a new node > 1.5 cm in any axis or a minimum of 1 cm in LDi of new extra-nodal lesions. All the PET/CT images were specifically reviewed by two nuclear medicine physicians (with at least 5 years of experience in PET/CT). In case of conflicting findings between the two observers, an independent panel of PET physicians with 10 years of experience in PET/CT would review the data and make the final decision.

### 2.3. Statistical Analysis

The current study assessed the consistency of ^18^F-FDG PET/CT and integrated CT results using the kappa consistency test. A *t*-test was used for continuous variables, and the χ^2^ test was used for categorical variables when assessing the associations between PET positivity and related clinical information. The significance of variables differentiating benign lesions from malignant FDG-avid lesions was evaluated by the logistic regression analysis. Survival probabilities were derived from Kaplan-Meier curves and compared using the log-rank test.

Statistical analyses were carried out using IBM SPSS Statistics (version 26.0). All tests were two-sided, and the value of *p* less than 0.05 was considered statistically significant.

## 3. Results

### 3.1. Patient Characteristics

The enrolled 185 patients (235 post-transplantation PET/CT scans) included 101 patients (105 post-transplantation PET/CT scans) with autologous SCT and 84 patients (130 post-transplantation PET/CT scans) with allogeneic SCT. Table 1 summarizes the demographic and clinical characteristics of the patients with autologous and allogeneic SCT. There were 167 NHL patients in total, including diffuse large B-cell lymphoma (*n* = 40), mantle cell lymphoma (*n* = 8), follicular cell lymphoma (*n* = 4), B lymphoblastic lymphoma (*n* = 10), Burkitt lymphoma (*n* = 4), small B-cell lymphoma (*n* = 1), anaplastic large cell lymphoma (*n* = 17), and peripheral T-cell lymphoma (*n* = 21), NK/T-cell lymphoma (*n* = 9), and T lymphoblastic lymphoma (*n* = 53).

The multivariate logistic regression analysis on 185 patients (235 post-transplantation PET/CT scans) was carried out. The results showed the integrated CT results, whether FDG-avid foci were in the sites of the original disease and Status at SCT were the significant factors for differentiating false-positive PET results from true-positive PET results (Table 2 and Table 3). However, due to a variety of differences between autologous and allogeneic SCT, we evaluated the two groups separately.

### 3.2. Autologous SCTs

#### 3.2.1. ^18^F-FDG PET-CT Results and Outcomes

Out of 105 post-transplantation PET/CT scans (101 patients), the presence of lymphoma foci was confirmed in 19 scans by pathological examination and follow-up practice. The sensitivity, specificity, positive predictive value (PPV), negative predictive value (NPV), and accuracy of post-SCT ^18^F-FDG PET-CT were 100% (19/19), 75.6% (65/86), 47.5% (19/40), 100% (65/65), and 80.0% (84/105), respectively, and those values of integrated CT were 84.2% (16/19), 84.9% (73/86), 55.2% (16/29), 96.1% (73/76), and 84.8% (89/105), respectively. The consistency of ^18^F-FDG PET-CT and integrated CT was moderate (Kappa = 0.424, *p* < 0.001). Among eight scans of positive CT results but negative PET results, all FDG-avid foci were demonstrated to be a benign process rather than lymphoma by pathological examination and follow-up practice.

**Figure 1:**
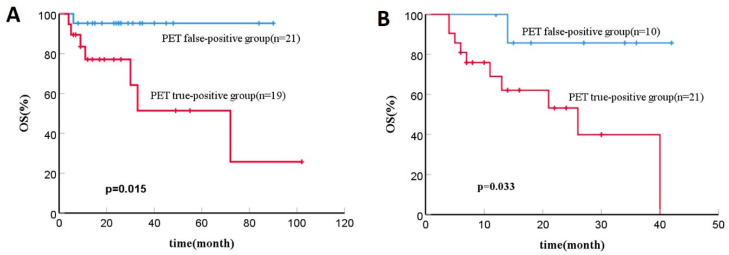
(**A**) Kaplan-Meier analysis of OS for lymphoma patients with 18F-FDG PET-CT findings after autologous SCT; (**B**) Kaplan-Meier analysis of OS for lymphoma patients with 18F-FDG PET-CT findings after allogeneic SCT.

On Kaplan-Meier analysis in patients with false-positive PET results, the 2-year OS rate was 95.2%, and the 5-year OS rate was 95.2%, in contrast to 77.1% and 51.4%, respectively, in those with true-positive PET results. (*p* = 0.015, Figure 1A).

#### 3.2.2. Analysis of Positive Results of Post-SCT ^18^F-FDG PET/CT

Table 2 shows the significance of variables in differentiating benign lesions from lymphoma-involved FDG-avid lesions. The results showed that false-positive cases were significantly more commonly seen when FDG-avid foci appeared outside the sites of the original disease (*p* = 0.004), and the integrated CT imaging showed negative results (*p* = 0.000). Multifocal foci were less common in patients with benign FDG-avid lesions than in those with lymphoma involvement, while the difference was not significant (*p* = 0.055).

Variables with *p* < 0.1 were taken into the regression analysis. In the multivariate logistic regression analysis, integrated CT results were the only significant factor for differentiating false-positive PET results from true-positive PET results (Table 3).

### 3.3. Allogeneic SCTs

#### 3.3.1. ^18^F-FDG PET-CT Results and Outcomes

Out of 130 post-transplantation PET/CT scans (84 patients), the presence of lymphoma foci was confirmed in 16.2% (21/130) scans by biopsy or follow-up imaging. The sensitivity, specificity, PPV, NPV, and accuracy of post-SCT ^18^F-FDG PET-CT were 100% (21/21), 90.8% (99/109), 67.8% (21/31), 100% (99/99), and 92.3% (120/130), respectively, and those values of integrated CT were 90.5% (19/21), 89.9% (98/109), 63.3% (19/30), 98.0% (98/100), and 90.0% (117/130), respectively. The consistency of ^18^F-FDG PET-CT and integrated CT was moderate (Kappa = 0.653, *p* < 0.001). Moreover, among seven scans of positive CT results but negative PET results, all FDG-avid foci were demonstrated to be a benign process rather than lymphoma by pathological examination or follow-up imaging.

A total of eleven patients died during the follow-up, including nine because of relapse or progression, one because of severe graft-versus-host disease (GVHD), and one due to serious infection. On Kaplan-Meier analysis in patients with false-positive PET results, the 2-year OS rate was 85.7%, and the 5-year OS rate was 85.7%, in contrast to 53.2% and 39.9%, respectively, in those with true-positive PET results. (*p* = 0.033, Figure 1B).

#### 3.3.2. Analysis of Positive Results of Post-SCT ^18^F-FDG PET/CT

**Figure 2:**
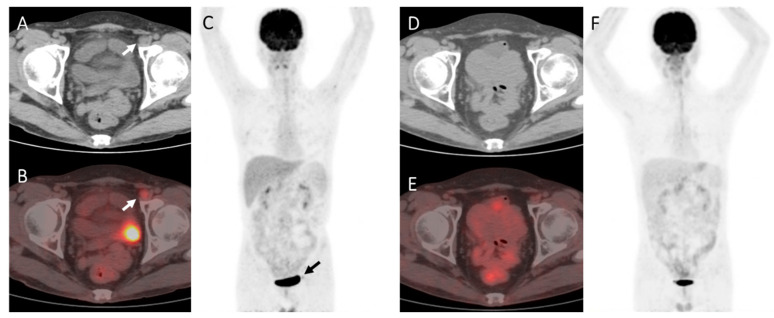
A case of B-Lymphoblastic lymphoma of the pancreas and bones in a 43-year-old male. The patient was treated with chemotherapy and achieved complete remission. The patient then received allogeneic SCT and underwent ^18^F-FDG PET-CT 1.5 months after transplantation for assessment. (**A**–**C**): PET showed an FDG-avid pelvic lymph node (arrow), about 1.3 * 1.1 cm^2^ in size, SUVmax3.5 (DS = 4). (**D**–**F**): A follow-up PET imaging was carried out 3 months later with no treatment, which showed the FDG-avid foci disappeared. Therefore the post-SCT PET at 1.5 months was determined as a false positive result.

**Figure 3:**
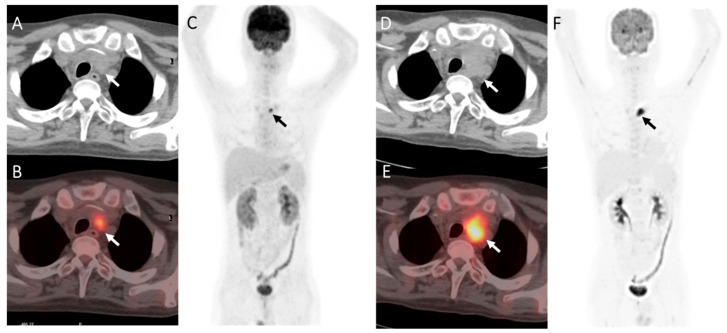
A case of T-Lymphoblastic lymphoma of the mediastinum in a 30-year-old male. The patient was treated with chemotherapy and achieved complete remission. The patient then received allogeneic SCT and underwent ^18^F-FDG PET-CT 5 months after transplantation for assessment. (**A**–**C**): PET showed an FDG-avid lesion in the upper mediastinum (arrow), about 1.7 * 1.2 cm^2^ in size, SUVmax7.61 (DS = 5). (**D**–**F**): A close follow-up PET imaging was carried out 1 months later with no treatment. It showed the upper mediastinum lesion (arrow) increased both in size (2.9 * 2.2 cm^2^) and ^18^F-FDG uptake (SUVmax10.85). Finally, it was clinically determined as a relapse.

Table 2 shows the significance of variables in differentiating benign lesions from lymphoma-involved FDG-avid lesions. The results showed that false-positive cases were significantly more commonly seen when DS = 4 but not 5 (*p* = 0.046), FDG-avid foci appeared outside the sites of the original disease (*p* = 0.022), and the integrated CT imaging showed negative results (*p* = 0.001) (Figure 2 and Figure 3). FDG-avid foci appearing on patients who received allo-SCT as the consolidation therapy after the first-line treatment seemed more likely to be false-positive, while the difference was not significant (*p* = 0.074) (Table 2).

Variables with *p* < 0.1 were taken into the regression analysis. In the multivariate logistic regression analysis, whether FDG-avid foci were in the sites of the original disease and integrated CT results were the significant factors for differentiating false-positive PET results from true-positive PET results (Table 3).

## 4. Discussion

Over the past several decades, PET has emerged as a critical approach for the accurate staging and restaging of lymphoma [25,26,27]. HSCT remains an important therapeutic option for lymphomas. However, after induction immunochemotherapy, increased ^18^F-FDG uptake is considered due to inflammation or incidental neoplasia rather than lymphoma in some lymphoma patients [28]. Normally, patients with autologous SCT often have a lower risk of infectious complications because they do not receive post-transplant immune suppression. Moreover, these patients do not develop GVHD because autologous SCT uses the patient’s own cells. On the other hand, the risk of disease relapse is often higher with autologous SCT when compared with allogeneic SCT. Allogeneic SCT recipients may develop acute and/or chronic GVHD, which results in immune-mediated cellular injury of several organs. Therefore, we evaluated the PET results after autologous or allogeneic SCT separately due to the differences. Ulaner’s study has confirmed that there is a high probability of false positives when interpreting PET imaging after allogeneic SCT but not autologous SCT, using the International Response Criteria (IRC) developed by the International Working Group in 2007 [18]. Qiao’s study demonstrated that FDG-PET findings after autologous SCT were true positive in eight out of ten FDG-positive patients with relapsed/refractory DLBCL [29]. Another study included a total of 41 patients with T-NHLs who underwent PET after autologous SCT for response assessment. The result showed that in thirteen nodal and eleven extra-nodal lesions, five lesions were false positive, but the other four lesions were ambiguous [30]. Our study showed that post-transplantation ^18^F-FDG PET-CT had not only a high sensitivity to detect lymphoma-involved lesions but also a high false-positive rate. However, we surprisingly found that the PPV of FDG PET-CT after autologous SCT and allogeneic SCT were 47.5% (19/40) and 67.8% (21/31), respectively, which means the false-positive FDG PET/CT was seen more frequently in patients with autologous SCT than patients with allogeneic SCT. This inconsistency requires further research to confirm.

In our study, Kaplan-Meier analysis showed that patients with false-positive PET results, as compared with those with true-positive PET results, were associated with a decreased rate of death. False-positive PET cases were significantly more commonly seen when the integrated CT imaging showed negative results. In addition, for the allogeneic SCT group, the possibility of false positives should also be considered when FDG-avid foci appeared outside the sites of the original disease. Noa Lavi [31] has proposed that the use of specific CT measurements can improve the PPV of surveillance ^18^F-FDG-PET/CT in patients with diffuse large B cell lymphoma (DLBCL). A study on lung cancer comes to similar conclusions that false-positive lymph nodes are associated with non-swollen nodes on ^18^F-FDG PET scans in lung cancer [32]. In recent years, effects of metabolic modulation by several new agents have been reported, therefore potentially increasing the incidence of false-positive ^18^F-FDG PET results [33] and mandating the emergence of “LYRIC” and “RECIL” criteria to consider tumor flare reactions [34,35]. It is suggested that both PET and CT image features need to be considered. For example, the criteria suggest that if the ^18^F-FDG uptake of one or more lesion(s) is increased without a concomitant increase in lesion size or number, it is mandatory to obtain a repeat imaging but not considered as true PD. However, it is mainly used for immunotherapy rather than SCT. Our results revealed that false-positive cases were significantly more commonly seen when the integrated CT imaging showed negative results (target nodal masses ≤ 1.5 cm in LDi or a new node ≤ 1.5 cm in any axis, or a new extra-nodal lesion ≤ 1 cm in LDi).

Most previous studies have used DS4 and DS5 as the PET-positive group when assessing treatment efficacy for lymphoma [36,37,38,39]. There are also studies suggesting that because patients with DS4 have a heterogeneous outcome, DS4 should be treated with caution [28,40,41]. In our present study, DS4 or DS5 was taken into the multivariate logistic regression analysis in the allogeneic SCT group, while the results showed that it was not a significant factor for differentiating false-positive PET results from true-positive PET results. In recent years, ΔSUV has been suggested as an indicator for efficacy evaluation. Rekowski J et al. [42] have considered that lymphoma patients with a relative reduction of the SUVmax between baseline and interim PET (iPET) staging of less than or equal to 66% have a poor prognosis. Xie W et al. [41] have proposed a modified-Deauville model which combines Deauville and ΔSUVmax methods. The results indicate that DLBCL patients with Deauville 4 and ΔSUVmax ≤ 70%, as well as those with Deauville 5, have a shorter OS. Boaz has suggested the end-of-treatment (EOT)/interim-metabolic volume (MV) ratio as a tool to identify patients at low risk of refractory disease, allowing non-invasive surveillance [43]. However, in our retrospective analysis, most patients did not have pre-transplantation PET or had salvage therapy between PET and transplantation. Therefore ΔSUVmax was not included in the study.

A recent study has divided DS5 into two situations: if ^18^F-FDG avidity of the original lesion markedly increased in the liver, it is defined as score 5a, and any new FDG-avid lesion score 5b. The result showed that at the end of treatment, the DS-5a score was highly suggestive of residual disease. However, participants with DS4 and DS-5b require histopathological confirmation before any change in treatment strategy to rule out false-positive [44]. Their time point of PET is after completing the first-line treatment program. Our results showed that for the allogeneic SCT group, the possibility of false positives should also be considered when FDG-avid foci appeared outside the sites of the original disease, suggesting that FDG-avid foci that appeared outside the sites of the original disease should be viewed with caution. One of our previous studies on acute leukemia patients treated with allo-SCT [45] has shown that FDG-avid lymph nodes >1.5 cm were not significantly associated with OS and DFS in multivariate analysis. This may be related to not considering other factors, such as if the FDG-avid foci appeared outside the sites of the original disease. However, prospective clinical studies with larger cohorts are required.

In this study, some patients underwent post-transplantation PET/CT more than once. This study included all PET examinations that met the inclusion criteria, not only the first PET examination after transplantation. The original intention was to observe if the false-positive rate of PET was related to the different time windows of post-SCT PET. However, the results showed that the false positive rate of PET within 3 months and within 3–6 months after autologous SCT was 10/20 and 11/20 (*p* = 1.00), respectively. The false positive rate of PET within 3 months and within 3–12 months after allogeneic SCT was 5/12 and 5/19 (*p* = 0.447), respectively. Undoubtedly, further research is needed to establish the optimal time window for post-SCT PET.

This study has certain limitations. First, the presence of lymphoma foci was confirmed in most cases by follow-up imaging but not biopsy when the post-SCT ^18^F-FDG PET/CT showed positive results. Second, other methods of PET/CT response assessment, such as ΔSUVmax or metabolic tumor volume, might provide different information from our current study. Furthermore, for the autologous SCT group, it was interesting to find that only CT results were predictable of PET/CT findings. Previous studies showed that multiple factors were associated with false or true positive PET results, such as status at SCT and DS score. This discrepancy might be caused by the lack of uniformity in the patient’s pathological type, treatment protocol, and the number of previous treatments. It is also another limitation of this study. However, it seemed to be certain that CT results played a significant role in determining whether the FDG-avid foci were disease recurrence.

## 5. Conclusions

Taken together, post-transplantation ^18^F-FDG PET/CT had a high sensitivity to detect lymphoma-involved lesions. However, false-positive ^18^F-FDG uptake in post-transplantation PET was not uncommon. When post-transplantation PET showed suspicious FDG-avid foci, integrated CT results were an important factor in identifying true or false positives in both autologous and allogeneic SCT cases. In addition, FDG-avid foci that occurred outside the sites of the original disease would likely be diagnosed as non-lymphoma involvement after allogeneic SCT.

## Figures and Tables

**Table 1:** Patient characteristics.

Characteristics	Autologous SCT	Allogeneic SCT
No. of patients	101	84
No. of post-transplantation PET/CT scans	105	130
Sex		
Male	65/101	53/84
Female	36/101	31/84
Age	37 (12–73)	28 (8–58)
Histology		
HL	16/101	2/84
B-NHL	48/101	20/84
T-NHL	37/101	62/84
Median follow-up, month (range)	24 (4–120)	16 (4–99)
Number of previous treatments		
1	50/101	33/84
>1	51/101	51/84
Status at SCT		
CR + PR	87/101	66/84
SD + PD	14/101	18/84
PET results		
Negative (DS < 4)	65/105	99/130
Positive (DS = 4 or 5)	40/105	31/130
Integrated CT results		
Negative	76/105	100/130
Positive	29/105	30/130
lymphoma involvement	19/105	21/130

SCT, hemopoietic stem cell transplantation; HL, Hodgkin’s lymphoma; NHL, non-Hodgkin’s lymphoma; CR, complete response; PR, partial response; SD, stable disease; PD, progressive disease; DS, Deauville score; PET, positron emission tomography; CT, computed tomography.

**Table 2:** Significance of clinical variables to differentiate false-positive and true-positive ^18^F-FDG PET studies for lymphoma involvement.

Variable	Autologous SCT			Allogeneic SCT			Autologous and Allogeneic SCT		
	False-Positive	True-Positive	*p* Value	False-Positive	True-Positive	*p* Value	False-Positive	True-Positive	*p* Value
**No. of positive post-transplantation PET/CT scans**	21	19		10	21		31	40	
**Sex**									
Male	12	13	0.527	5	13	0.701	17	26	0.465
Female	9	6		5	8		14	14	
**Age**	31 (13–66)	37 (18–59)	0.506	44 (8–46)	29 (13–58)	0.102	37 (8–66)	34 (13–59)	0.431
**Histology**									
HL	5	3	0.546	0	1	0.615	5	4	0.226
B-NHL	9	6		4	5		13	11	
T-NHL	7	10		6	15		13	25	
**Number of previous treatments**									
1	9	6	0.527	5	3	0.074	14	9	0.072
>1	12	13		5	18		17	31	
**Status at SCT**									
CR + PR	19	16	0.654	10	15	0.141	29	31	0.098
SD + PD	2	3		0	6		2	9	
**Conditioning regimen (auto-SCT)**									
BEAM	14	14	0.886	-	-	-	-	-	-
BuCy	4	2		-	-				
others	3	3		-	-		-	-	
**Conditioning regimen (allo-SCT)**									
BuCy	-	-	-	6	11	1.000	-	-	-
TBI containing treatment	-	-		4	10		-	-	
**Donor type**									
HLA identical sibling	-	-	-	1	6	0.193	-	-	-
HLA haploidentical sibling	-	-	-	8	9		-	-	
Unrelated	-	-	-	1	6		-	-	
**Timing of Post-SCT ^18^F-FDG PET-CT**									
Within 3 months	10	10	1.000	5	7	0.447	-	-	-
Within 3–6 months (auto-SCT)	11	9					-	-	
Within 3–12 months (allo-SCT)				5	14		-	-	
**Immunomodulatory or CAR-T therapy prior to post-SCT PET scan**									
Yes	0	1	0.475	0	3	0.533	0	4	0.126
No	21	18		10	18		31	36	
**DS score**									
DS = 4	16	9	0.102	9	10	0.046 *	25	19	0.006 *
DS = 5	5	10		1	11		6	21	
**Unifocal or multifocal**									
Unifocal	15	7	0.055	7	7	0.121	22	14	0.004 *
Multifocal	6	12		3	14		9	26	
**FDG-avid foci occurred in the sites of original disease**									
Yes	10	1	0.004 *	5	2	0.022 *	15	3	0.000 *
No	11	18		5	19		16	37	
**FDG-avid foci were in lymph nodes only**									
Yes	16	13	0.723	5	7	0.447	21	20	0.153
No	5	6		5	14		10	20	
**Integrated CT results**									
Negative	18	1	0.000 *	7	2	0.001 *	25	3	0.000 *
Positive	3	18		3	19		6	37	
**Median follow-up, month (range)**	26 (6–90)	17 (4–102)	0.015 *	12 (7–42)	14 (4–40)	0.033 *	25(6–90)	14 (4–102)	0.000 *

* *p* < 0.05; SCT, hemopoietic stem cell transplantation; HL, Hodgkin’s lymphoma; NHL, non-Hodgkin’s lymphoma; CR, complete response; PR, partial response; SD, stable disease; PD, progressive disease; FDG, fluorodeoxyglucose; DS, Deauville score; PET, positron emission tomography; CT, computed tomography; BEAM, carmustine, etoposide, cytarabine, and melphalan; BuCy, busulfan and cyclophosphamide; TBI, total body irradiation; HLA, Human Leucocyte Antigen; CAR-T, chimeric antigen receptor T cells.

**Table 3:** Multivariate logistic regression analysis to identify clinical variables to differentiate false-positive and true-positive ^18^F-FDG PET studies for lymphoma involvement.

Variable	Autologous SCT		Allogeneic SCT		Autologous and Allogeneic SCT	
	HR (95%CI)	*p*-Value	HR (95%CI)	*p*-Value	HR (95%CI)	*p*-Value
**Number of previous treatments**	-	-	-	0.626	-	0.106
**Unifocal or multifocal**	-	0.504	-	-	-	0.380
**FDG-avid foci occurred in the sites of original disease**	-	0.079	19.706 (1.347–288.297)	0.029 *	14.849 (2.324–95.469)	0.004 *
**DS score**	-	-	-	0.353	-	0.361
**Integrated CT results**	108.000 (10.243–1138.782)	0.000 *	38.741 (3.028–495.745)	0.005 *	151.307 (14.646–1563.120)	0.000 *
**Status at SCT**	-	-	-	-	54.583 (2.182–1365.641)	0.015 *

* *p* < 0.05; SCT, stem cell transplantation; FDG, fluorodeoxyglucose; DS, Deauville score; CT, computed tomography.

## Data Availability

The datasets used and/or analyzed during the current study are available from the corresponding author on reasonable request.
